# Molecular Mechanisms of Adaptation of the Moderately Halophilic Bacterium *Halobacillis halophilus* to Its Environment 

**DOI:** 10.3390/life3010234

**Published:** 2013-02-27

**Authors:** Inga Hänelt, Volker Müller

**Affiliations:** Department of Molecular Microbiology and Bioenergetics, Goethe University Frankfurt am Main, Max-von-Laue-Str. 9, 60438 Frankfurt am Main, Germany; E-Mail: haenelt@bio.uni-frankfurt.de

**Keywords:** osmoadaptation, chloride accumulation, compatible solutes, hybrid strategy

## Abstract

The capability of osmoadaptation is a prerequisite of organisms that live in an environment with changing salinities. *Halobacillus halophilus* is a moderately halophilic bacterium that grows between 0.4 and 3 M NaCl by accumulating both chloride and compatible solutes as osmolytes. Chloride is absolutely essential for growth and, moreover, was shown to modulate gene expression and activity of enzymes involved in osmoadaptation. The synthesis of different compatible solutes is strictly salinity- and growth phase-dependent. This unique hybrid strategy of *H. halophilus* will be reviewed here taking into account the recently published genome sequence. Based on identified genes we will speculate about possible scenarios of the synthesis of compatible solutes and the uptake of potassium ion which would complete our knowledge of the fine-tuned osmoregulation and intracellular osmolyte balance in *H. halophilus*.

## 1. Introduction

Salt marshes are costal ecosystems in the upper intertidal zone between land and open sea water. Soil and water of this area face drastic changes in salinities since the land is regularly flooded by tides. In addition, water evaporates in summer leading to dryness and extremely high salinities of up to 3 M NaCl, while extensive rainfalls can decrease the salinity to fresh water concentrations. Organisms from all kingdoms of life that live in these areas have to adapt to such changing salinities by various strategies of osmoadaptation. 

A well-studied model organism for osmoadaptation is the rod-shaped, endospore-forming, Gram-positive bacterium *Halobacillus halophilus*, which was isolated from a salt marsh on the North Sea coast of Germany and was originally described as *Sporosarcina halophila* [1]. Based on 16S rRNA homologies, it is now phylogenetically classified within the order *Bacillales*, Class *Bacilli*, Phylum *Firmicutes* and has been renamed to *Halobacillus halophilus* [2]. Being moderately halophilic, *H. halophilus* grows optimally between 0.5 and 2.0 M NaCl but can tolerate NaCl concentrations of up to 3.0 M NaCl with a growth rate of 38% of the optimum [3]. Strategies of osmoadaption of *H. halophilus* have been studied extensively in the past decades demonstrating a highly salinity- and growth phase-dependent adaption. This review will summarize the molecular mechanisms of osmoadaption in context of the recently published genome.

## 2. Hybrid Strategy for Long-Term Adaptation to Saline Environments

In general, two strategies are known to cope with changing or constantly high salinities of the environment. Organisms that grow optimally in the presence of extremely high salinities of up to 5 M NaCl, termed halophiles, accumulate intracellular KCl in concentrations higher than the external NaCl concentration to maintain a turgor pressure. This so called ‘salt-in’ strategy is found in the *Halobacteriales* (archaea) and the bacterium *Salinibacter ruber* [4,5]. Cellular processes and machineries of organisms following this ‘salt-in’ strategy are adapted to high internal KCl, such that in general these halophiles are restricted to growth at high salt. A more flexible strategy is found in moderately halophilic bacteria that grow over a wide range of salinities (typically 0.5−1 to 3 M NaCl) [6]. This strategy, the ‘low-salt-in’ strategy, relies on the accumulation of high concentrations of organic compatible solutes. Compatible solutes are small, mainly neutral but polar compounds (sugars, amino acids and derivates as well as polyols) which are highly soluble in water and do not interfere with the cellular metabolism. Thus, other than for KCl, a broad variation of the intracellular concentration of those compounds is possible without effecting cellular processes. The uptake or synthesis of compatible solutes retains a cytoplasm iso-osmotic with or slightly hyperosmotic compared to its surroundings.

*H. halophilus* has originally been described as a bacterium that amasses compatible solutes to establish a cellular turgor [2]. However, later it was shown that it also accumulates molar concentrations of chloride in the cytoplasm [3]. This survival strategy is now seen as a unique hybrid strategy of the moderately halophilic *H. halophilus* to cope with changing salinities of the environment. 

The hypothesis of this hybrid strategy arose from the fact that *H. halophilus* amasses compatible solutes as well as chloride in the cytoplasm as we will discuss below. However, similar conclusions resulted from the recently published analysis of the proteome of *H. halophilus* deduced from the genome sequence [7]. Comparisons of different genome sequences revealed a clear distinction of proteomes from extreme halophiles and non-halophiles with a higher number of acidic proteins in the extreme halophiles. Within this comparison, the proteome of *H. halophilus* takes an intermediate position as the averaged isoelectric point of all proteins of 6.6 is slightly acidic. This trend was seen for soluble as well as for membrane proteins and supports an intermediate strategy in osmoadaptation [7]. 

## 3. The Chloride Modulon

**Figure 1:**
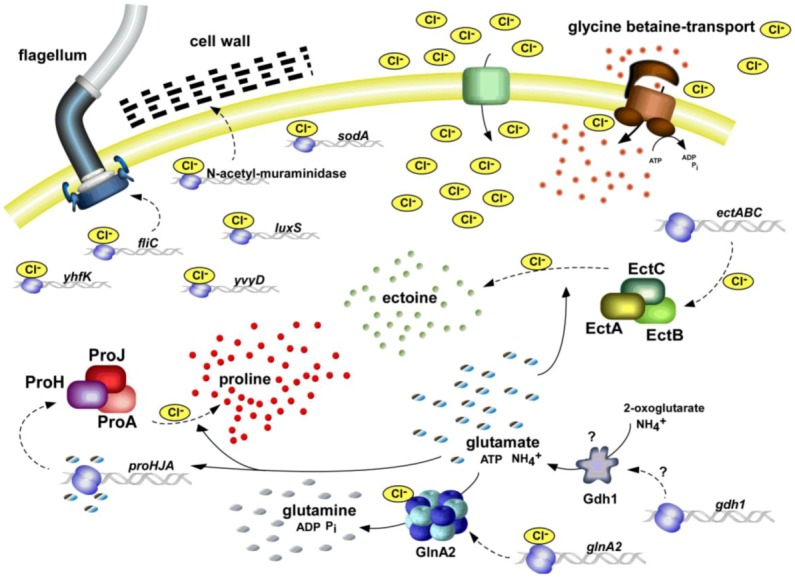
The chloride regulon of *Halobacillus halophilus*. All known processes that were identified to be influenced by the presence of chloride are summarized. Both, on transcriptional and enzyme activity level, chloride was found to have stimulating effects [10]. For further explanations see text.

One of the outstanding, or even to our knowledge, unique physiological features of *H. halophilus* is its chloride dependence of growth, gene expression and enzymatic activity. Growth of *H. halophilus* was shown to strictly depend on the presence of chloride, in absence of chloride the strain does not grow. As a consequence of high external salinities, Cl^−^ was shown to accumulate in the cytoplasm following the ‘salt-in’ strategy. While the internal Cl^−^ concentration is negligible at low external Cl^−^ concentrations, it increases to 50% of the external Cl^−^ concentration at higher salt concentrations [3]. Moreover, *H. halophilus* not only depends on Cl^−^ to compensate for increasing external salinities but Cl^−^ also regulates cellular processes. Increasing external chloride concentrations and not the salinity in general were determined to regulate the germination of endospores [8] and the motility of the vegetative cell [9] but also to control the expression of genes and the enzymatic activity of proteins needed for the halophilic life style of *H. halophilus* [10]. Most of these regulated proteins are key players of the biosynthesis of compatible solutes which are essential to cope with high and changing salinities. All known processes regulated by chloride are summarized in the chloride modulon (Figure 1). 

An open question is how chloride accumulates in the cytoplasm. Since the measured internal chloride concentrations are too high to be in equilibrium with the membrane potential, it has been suggested that chloride is actively accumulated into the cytoplasm of *H. halophilus* [3]. However, so far no gene could be identified to encode for a chloride transporter or even a simple channel [7]. Several genes are annotated to encode for potential symporters but their substrate specificity remains to be elucidated. To completely understand the chloride modulon, the chloride transporter has to be found and characterized in detail.

Also the counterion for chloride is not known but is likely to be potassium as shown for many other organisms. However, it is not yet understood why potassium is the main monovalent intracellular cation and why it is preferred compared to Na^+^ in this function. In bacteria, K^+^ might modulate activity and correct folding of proteins more effectively than Na^+^. Another explanation for the accumulation of K^+^ and the concomitant active extrusion of Na^+^ is that this situation enables the cells to establish an inwardly directed electrochemical transmembrane Na^+^ gradient, which in consequence is used for energy-consuming processes like secondary transport or flagella movement [11]. Interestingly, the genome of *H. halophilus* encodes for Ktr-type potassium transporters (Hbhal_1246, Hbhal_2758, Habl_4548) and two potassium channels (Hbhal_3837, Hbhal_3881) only while high-affinity, ATP-dependent transporters like the Kdp system were not identified [7]. Further studies are needed to elucidate the role of potassium and identify involved uptake systems. 

## 4. Biosynthesis of Compatible Solutes

**Figure 2:**
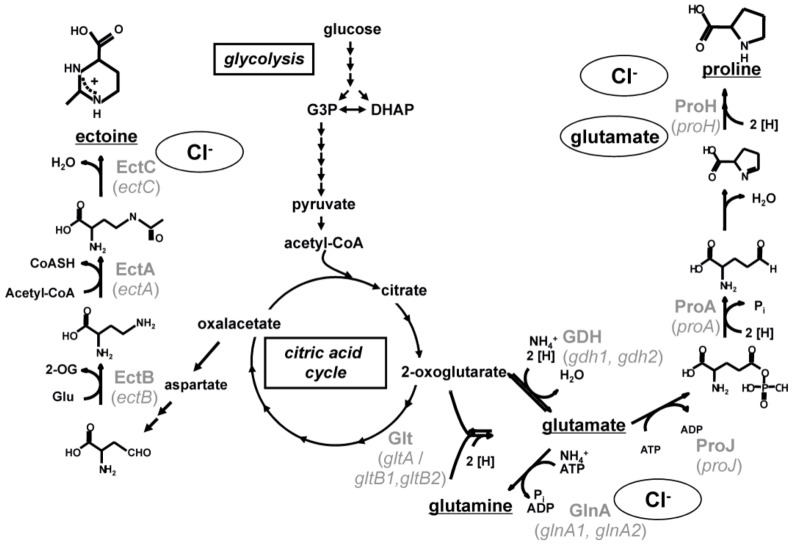
Proposed biochemical pathways of the main compatible solutes in *Halobacillus halophilus*. The pathways for the synthesis of glutamate, glutamine, proline and ectoine are shown in black, the involved enzymes (and coding genes) in gray. Stimulating effects of chloride and glutamate on gene transcription and enzyme activity are indicated [10].

Next to accumulating internal Cl^-^, *H. halophilus* synthesizes a cocktail of different solutes to combat external salinity. The major solutes are glutamate, glutamine and proline but also ectoine, N^δ^-acetyl ornithine and N^ε^-acetyl lysine are produced [7,10]. The biosynthesis pathways of glutamate, glutamine, proline and ectoine are predicted based on the genome sequence and studied biochemically (Figure 2) while the pathways of N^δ^-acetyl ornithine and N^ε^-acetyl lysine are deduced from the genome sequence only.

Three main enzymatic reactions are known for the biosynthesis of glutamate and glutamine [12]. The biosynthesis of glutamate can either be accomplished by the action of a sequence of glutamine synthetase and glutamate synthase (GOGAT) or by the action of a glutamate dehydrogenase (GDH) while glutamine is synthesized by a glutamine synthetase (Gln) only. The genome contains two putative open reading frames encoding a glutamate synthase (*gdh1* and *gdh2*), only one open reading frame encoding the large subunit of a glutamate synthase (*gltA*) and two open reading frames each potentially encoding the small subunit of a glutamate synthase (*gltB1* and *gltB2*). The glutamine synthetase Gln is encoded by two open reading frames (*glnA1* and *glnA2*). While the former (*glnA1*) clusters with a gene (*glnR*) encoding the regulatory protein GlnR which is known to be essential in nitrogen metabolism from *Bacillus subtilis*, the latter (*glnA2*) lies solitary and is predicted to be regulated by a promotor recognized by σ^B^, the general stress σ-factor.

Proline is synthesized from glutamate by a sequence of a glutamate 5-kinase (ProJ), a glutamate 5-semialdehyde dehydrogenase (ProA) and a pyrroline-5-carboxylate reductase (ProH) [13]. The enzymes are encoded by a cluster of three genes—*proH, proJ* and *proA*, which are organized in an operon.

Also the three biosynthetic genes (*ectABC*) for the production of ectoine are arranged on one operon [14]. Ectoine is produced from aspartate semialdehyde by a sequence of a diaminobutyrate-2-oxoglutarate transaminase (EctB), a diaminobutyric acid acetyltransferase (EctA) and an ectoine synthase (EctC).

**Figure 3:**
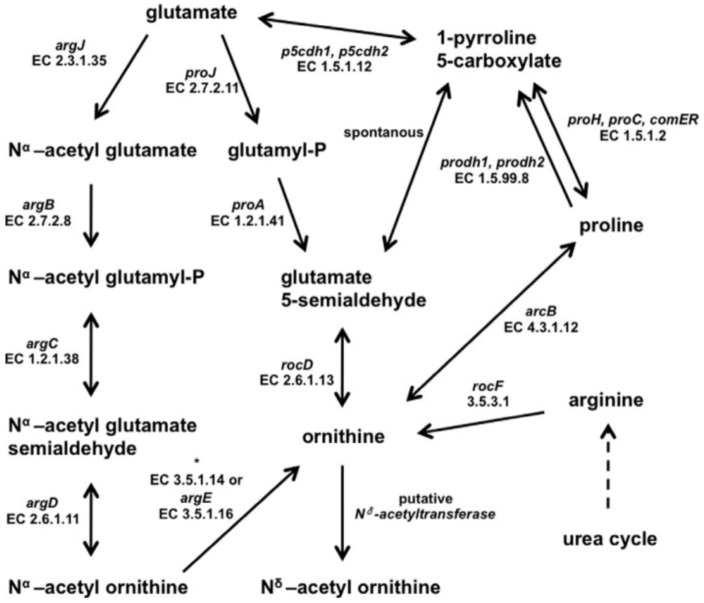
Putative biosynthetic pathways of *N*^δ^-acetyl ornithine. Most likely, *N*^δ^-acetyl ornithine is formed from ornithine catalysed by a so far unidentified *N*^δ^-acetyltransferase. Based on the genome sequence, the pool of ornithine could be replenished by the conversion of glutamate, proline or arginine catalysed by the reactions depicted in the diagram. The asterisk ‘*’ indicates genes for which no gene name has yet been assigned [7].

The pathway for the production of N^δ^-acetyl ornithine still has to be elucidated but presumably ornithine is the direct precursor, for which several pathways are known. All necessary genes encoding the corresponding enzymes were identified in the genome of *H. halophilus* and possible pathways were described in detail by Saum and colleagues recently (Figure 3) [7].

The last potential compatible solute that is synthesized by *H. halophilus* is N^ε^-acetyl lysine. However, its role as compatible solute still has to be confirmed by further studies. Based on the now available genome sequence it was assumed that *H. halophilus* is capable of synthesizing lysine by use of the classical diaminopimelate pathway [7]. In this pathway aspartate is initially activated by an aspartate kinase. *H. halophilus* possesses two copies of this enzyme (*dapG1*, Hbhal_3090, *dapG2*, Hbhal_3465) which might be, similar to the glutamine synthetase, subject to different modes of regulation. The activated aspartyl moiety in consequence gets reduced to the corresponding semialdehyde [catalysed by an aspartate semialdehyde dehydrogenase (*asd*, Hbhal_3089)] which consequently undergoes a condensation reaction with one molecule of pyruvate resulting in the formation of 2,3-dihydrodipicolinate. This reaction, which is the first that differs from the ectoine biosynthesis pathway, is catalyzed by the dihydrodipicolinate synthase. Three copies of the corresponding gene were identified (*dapA1*, Hbhal_2387, *dapA2*, Hbhal_3091, *dapA3*, Hbhal_5017) which may indicate its critical role in the biosynthesis. It is likely that the different genes are controlled by different demands such as the need for lysine as a compound in protein biosynthesis, the need of N^ε^-acetyl lysine as osmoprotectant or the need to provide precursors for the biosynthesis of peptidoglycan. The following sequence of reactions leading to lysine is then catalyzed by a dihydrodipicolinate reductase (Hbhal_3261), a tetrahydrodipicolinate N-acetyltransferase (Hbhal_2790), an acetyltransferase, an N-acetyldiaminopimelate deacetylase (Hbhal_2791), a diaminopimelate epimerase (*dapF*, Hbhal_2627) and finally a diaminopimelate decarboxylase (*lysA*, Hbhal_3343). The final acetylation at the ε-amino group requires an acetyltransferase (Hbhal_3877).

## 5. Salinity- and Growth Phase-dependent Adaptation of the Solute Pool

**Figure 4:**
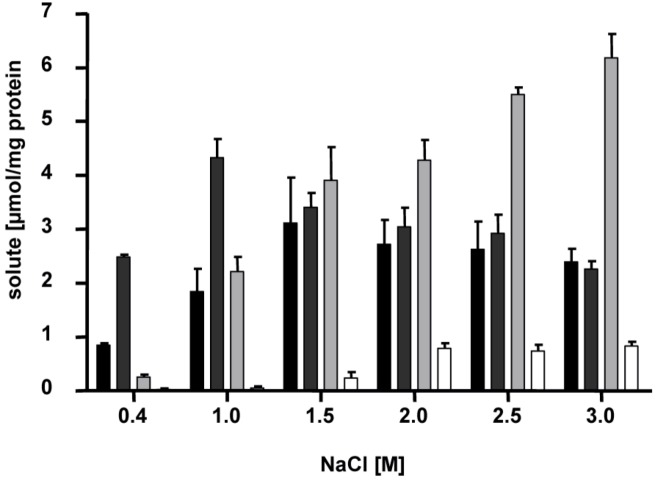
Accumulation of glutamine, glutamate, proline and ectoine is dependent on the NaCl concentration of the medium. Cells of *H. halophilus* were cultivated in mineral salt medium (G10) in the presence of the NaCl concentrations indicated. They were harvested in the exponential growth phase (OD_578_. 0.6 to 0.8), compatible solutes were extracted, and the concentrations of glutamine (black), glutamate (dark gray), proline (light gray) and ectoine (white) were measured by HPLC [14].

Interestingly, the compatible solutes listed above do not appear all at the same time but *H. halophilus* switches its osmolyte content depending on salinity (Figure 4) [10]. At intermediate salinities of around 1.5 M NaCl, glutamate and glutamine are the major solutes. Transcription analyses after an osmotic upshock from 0.8 to 2 M NaCl have shown that one of the putative glutamate dehydrogenase genes (*gdh1*) was induced and the mRNA level increased within 1.5 hours to about four-fold compared to the level before the upshock. This enables an increased production of glutamate from 2-oxoglutarate and NH^4+^. In contrast, the transcript levels of the second glutamate dehydrogenase gene (*gdh2*) were close to the detection limit likely being involved in nitrogen metabolism rather than osmoregulation. So far, also the glutamate synthase gene (*gltA*) did not seem to be involved in osmoregulation. Glutamine is synthesized by the action of a glutamine synthetase which is encoded by two genes (*glnA1* and *glnA2*) in *H. halophilus*. On a transcriptional level only the expression of *glnA2* was shown to be upregulated at increasing salt concentrations with a maximal increase of transcripts of about 4-fold (compared to the value at 0.4 M NaCl) at 1.5 M NaCl or higher. The expression of *glnA1* was not affected. Moreover, the expression of *glnA2* and especially the glutamine synthetase activity were shown to be chloride-dependent being increased with increasing chloride concentrations in the surrounding media. The maximal enzymatic activity was found at 2.5 M NaCl or higher [12]. This is in line with the chloride dependence of growth and the accumulation of Cl^-^ into the cytoplasm as described above. However, it is unknown how chloride modulates the enzymatic activity. Both, a direct interaction of chloride with the glutamine synthetase or the participation of a regulatory protein that senses and mediates the concentration of chloride, are possible. 

At high salinities (2.0 M NaCl or higher), glutamine and glutamate pools stay rather constant but proline is produced in addition, and becomes the dominant solute. It was shown that the transcription of the *pro* operon was increased with increasing salinities with a maximum at 2.5 M. The mRNA level reached a maximum 1.5 hours after an osmotic upshock while the maximal concentration of proline was determined after 6 hours. Consequently, the increased amount of enzymes led to an increased production of proline [13]. However, not only NaCl but also Na-glutamate had an effect on gene expression. Compared to NaCl Na-glutamate was shown to dramatically increase the *proHJA* mRNA concentration. Since proline is produced from glutamate and Na-glutamate had a more stimulating effect on gene expression than NaCl, NaCl now is supposed to be the initial signal for the proline production only. Glutamate instead acts as ‘second messenger’ that further regulates the pro operon expression by its internal concentration which increases with increasing salinity [13]. 

In addition to the salinity-dependent solute regulation, another layer of regulation is active: proline contents are maximal in exponentially growing cultures but reduced in stationary phase cultures. Under these conditions, ectoine is synthesized. To resolve the time-dependent kinetics of ectoine production *H. halophilus* cells were subjected to an osmotic upshock from 0.8 to 2.0 M NaCl and the biosynthesis of ectoine was measured at the levels of transcription, translation and solute accumulation. Transcripts were readily detectable directly after the upshock, but increased dramatically with time and reached a maximum 3 hours later. Most important, the expression of ect genes was preceded by expression of genes responsible for glutamine, glutamate or proline biosynthesis. The signal leading to *ect* gene transcription is therefore assumed to be an indirect one mediated by one or more yet to be identified factors rather than by the presence of the osmolyte. The production of the ectoine synthase EctC nicely corresponds to the increase of *ectC* transcript. Both were found to increase 2- fold. Surprisingly, 4 hours after upshock the EctC concentration again decreased with time and the level reached a value only slightly above the value at the beginning, although the external stress was still present. This decrease, however, was not reflected in the ectoine concentration, which steadily increased and reached a maximum 18 hours after upshock. Again, this demonstrates a great delay in accumulation compared to proline that reached its maximum already 6 hours after upshock and hints to a role of ectoine not only in the immediate response to osmotic upshock but to a function as a more general protectant in the cell [14]. 

To quickly adapt to changing salinities and growth phases *H. halophilus* regulates the synthesis of solutes by both increasing expression of the enzymes involved and activating the produced enzymes in a chloride-dependent manner. *H. halophilus* also has a gene encoding a potential ectoine hydroxylase, but hydroxyectoine has not been detected yet [7]. *H. halophilus* does not have the genetic capacity for *de novo* biosynthesis of glycine betaine, but can take up choline from the environment and oxidize it to glycine betaine [15]. 

## 6. Catabolic Traits and Nutritional Versatility

The observed preference of specific compatible solutes is also reflected by possible catabolic traits and nutritional versatility of *H. halophilus*. *H. halophilus* is a chemoorganoheterotrophic, strictly aerobic bacterium with great nutritional versatility. It is able to hydrolyze complex substrates such as casein, gelatin, DNA, starch and pullulan [1]. Genes encoding two extracellular proteases (Hbhal 5155 and Hbhal 4449), one amylase (Hbhal 4101) and one pullulanase (Hbhal 2962) were identified in the genome. In addition, *H. halophilus* is able to grow on hexoses such as glucose or fructose (by way of glycolysis) and on amino acids. Noteworthy is the use of carbon sources that are also used as compatible solutes such as glutamate and proline. Proline and glutamate, major compatible solutes of *H. halophilus*, are good growth substrates and may also be used as nitrogen sources, indicating a sophisticated regulatory network balancing different cellular needs. For example, proline is degraded by proline dehydrogenase (ProDH) and Δ^1^-pyrroline-5-carboxylate dehydrogenase (P5CDH) to glutamate via Δ^1^-pyrroline-5-carboxylate of which *H. halophilus* has two isogenes each for *prodh* and *p5cdh*. *prodh2* and *p5cdh2* form an operon (put operon) that is involved in the utilization of proline as carbon and energy source whereas ProDH1 and P5CDH1 may be involved in supplying the cell with nitrogen from proline [7]. 

In contrast to *Halomonas elongata*, the genome of *H. halophilus* does not encode for ectoine utilization genes, consistent with the observation that ectoine is only a minor solute in *H. halophilus*.

## 7. Concluding Remarks

*H. halophilus* is the first moderate halophilic bacterium shown to use a hybrid strategy for osmoadaptation by accumulating both molar concentrations of chloride and compatible solutes. This unique feature enables *H. halophilus* to grow over a broad range of salinities and to adapt sufficiently to rapidly changing environments. Other than hyperosmotic organisms, *H. halophilus* can survive at low NaCl concentrations but also copes with relatively high salt concentrations of up to 3 M. The sophisticated salinity- and growth phase-dependent adaptation of the accumulated solutes is remarkable and probably demonstrates a long lasting evolution being optimally prepared for its changing environment. This adaptation is also reflected by the use of a dominant compatible solute, such as carbon and nitrogen, to guarantee energy optimization. 
